# Oral malignant melanoma detected after resection of amelanotic pulmonary metastasis^☆^

**DOI:** 10.1016/j.ijscr.2013.10.004

**Published:** 2013-10-17

**Authors:** Katsunari Matsuoka

**Affiliations:** Department of Thoracic Surgery, National Hospital Organization Himeji Medical Center, 670-8520 Honmachi 68, Himeji-City, Hyogo, Japan

**Keywords:** Malignant melanoma, Oral cavity, Pulmonary metastasis

## Abstract

**INTRODUCTION:**

Solitary pulmonary metastasis from oral malignant melanoma is very rare.

**PRESENTATION OF CASE:**

We demonstrated a 84-year-old patient with a lung nodule that was diagnosed as malignant melanoma by video-assisted thoracoscopic resection. Because primary pulmonary malignant melanoma was extremely rare, the tumor was thought to be a metastasized from an occult primary lesion. A detailed physical examination revealed a black tumor in the oral cavity, and this was suspected to have been the primary. Resection of the hard palate tumor and dissection of the cervical lymph nodes were performed. The patient was simply followed up without further therapy at his request, and he died one year after surgery due to bleeding from a pleural metastasis of malignant melanoma.

**DISCUSSION:**

Primary melanoma of the oral cavity is rare, accounts for 0.5% of all oral cancers, and 0.8–1.8% of all melanomas. Because of absence of symptoms in the early stage of the disease and the presence of the tumor in relatively obscure areas of the oral cavity, the diagnosis is unfortunately often delayed. In view of the rarity of primary lung melanoma, when lung tumor was diagnosed as malignant melanoma, detailed physical examination of the entire skin and mucosa including the oral cavity was necessary.

**CONCLUSION:**

Oral malignant melanoma was very rare, but oral cavity should be examined when the pulmonary nodule was diagnosed as malignant melanoma.

## 1 Introduction

Primary melanoma of the lung is exceptionally rare. We presented a resected case of malignant melanoma of the lung that was diagnosed as metastatic tumor from an oral malignant melanoma after post-surgical physical examination including the oral cavity.

## 2 Presentation of case

**Fig. 1:**
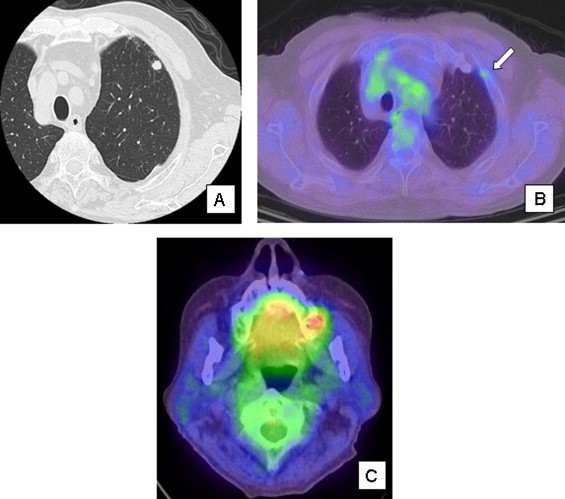
Chest CT on admission demonstrated the nodular lesion of 8 mm in diameter in the left upper lobe of lung. (A) Positron emission tomography demonstrated slight uptake of FDG at the position of the nodular shadow (white arrow) and no other abnormal findings were evident. Although FDG uptake of left cervical lymph node was increased, this uptake was supposed to nonspecific uptake due to nonspecific lymph node swelling (B) and (C).

**Fig. 2:**
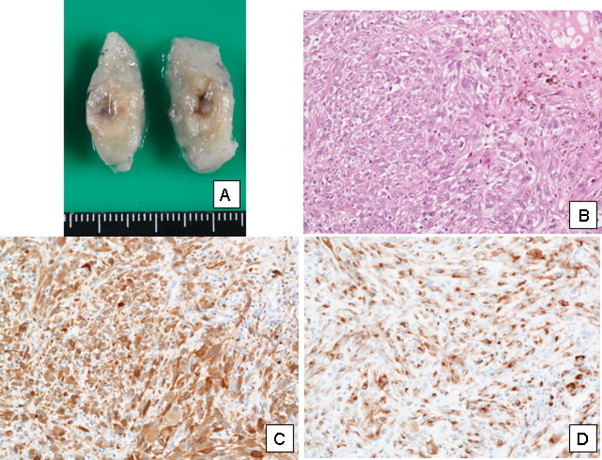
The resected nodule had a smooth surface, and the cut surface was white with brown pigmented deposits (A). Histological examination demonstrated a proliferation of spindle and epithelioid cells with abundant cytoplasm and atypia (B, H-E stain 200×). Immunohistochemical examination demonstrated positively for S100 and HMB-45 (C: S100 200×, D: HMB45 200×).

An asymptomatic 84-year-old male was presented at our hospital because a nodular lesion of 8 mm in diameter had been detected in the left upper lobe of lung by routine chest computed tomography during follow up for ischemic heart disease (Fig. 1). He had undergone subtotal gastrectomy for early gastric cancer, radiation treatment and hormone therapy for prostate cancer, and coronary artery stenting due to severe multiple coronary artery stenosis. He had a history of smoking with a pack-year rate of 35. Retrospective examination revealed a 6 mm nodular lesion on a chest CT film obtained 4 months previously. Positron emission tomography demonstrated only slight uptake of FDG at the position of the nodular shadow, and no other abnormal findings were evident (Fig. 1). Although FDG uptake of left cervical lymph node was increased, this uptake was supposed to nonspecific uptake due to nonspecific lymph node swelling. Because the nodule increased in size during 4 months, malignant disease was suspected, and we performed video-assisted thoracoscopic surgery. Because of the patient's poor cardiac function, wedge resection was performed. The resected nodule had a smooth surface, and the cut surface was white with brown pigmented deposits. Intraoperative histological examination gave a diagnosis of undifferentiated carcinoma. The postoperative course was uneventful, and the patient was discharged from hospital 3 days after surgery. Histological examination demonstrated a gray-white tumor with brown spots and proliferation of spindle and epithelioid cells with abundant cytoplasm and atypia. The tumor involved the visceral pleura and was exposed to the pleural surface histologically. Immunohistochemical examination demonstrated negatively for CAM5.2, CK7, TTF-1, NapsinA and calretinin, and positively for vimentin, HMB-45 and S100. From these results, the tumor was diagnosed as malignant melanoma (Fig. 2).

**Fig. 3:**
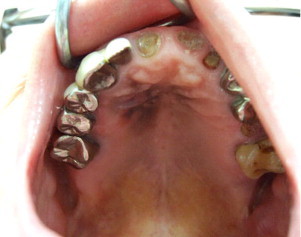
Slightly elevated black lesions with irregular boundaries approximately 20 mm in diameter was detected on the palatal mucosa.

Because primary pulmonary malignant melanoma was extremely rare, the tumor was thought to be a metastasized from an occult primary lesion. A whole skin examination showed no abnormal findings. However, examination in oral cavity revealed slightly elevated, black lesions with irregular boundaries approximately 20 mm in diameter on the palatal mucosa (Fig. 3). The patient had not been aware of this lesion. An incisional biopsy was performed under local anesthesia, and the tumor was diagnosed as malignant melanoma. Resection of the hard palate tumor and dissection of the cervical lymph nodes were performed at the department of otolaryngology of our institute. Although the melanoma was at an advanced stage, the patient was simply followed up without further chemotherapy or immunotherapy at his request, and he died one year after surgery due to bleeding from a pleural metastasis of malignant melanoma.

## 3 Discussion

We have described a patient with a lung nodule that was diagnosed as malignant melanoma by histological examination. Because primary melanoma of the lung is exceptionally rare, only about 30 cases having been reported in English literature, and the lung is the most common site of metastasis from malignant melanoma,^1^ the present lung tumor was thought to have metastasized from an occult primary tumor. A detailed physical examination revealed a black tumor in the oral cavity, and this was suspected to have been the primary. Shimmyo et al. also demonstrated a case of malignant melanoma that primary lesion was detected 8 month after resection of lung metastasis.^2^ In view of the rarity of primary lung melanoma, physical examination of the entire skin and mucosa, including the oral cavity, was necessary. Although positron emission tomography (PET) scanning is useful for detecting malignant diseases, we were unable to detect the primary tumor by PET preoperatively in this case. PET scan detected the lung tumor but not the oral tumor. Even if PET does not demonstrate abnormal uptake, detailed examination of the entire body is necessary.

Oral mucosal melanoma is a rare neoplasm. Primary melanoma of the oral cavity accounts for 0.5% of all oral cancers, and 0.8–1.8% of all melanomas.^3^ Because of absence of symptoms in the early stage of the disease and the presence of the tumor in relatively obscure areas of the oral cavity, the diagnosis is unfortunately often delayed. Aggressive resection with complete removal of the tumor is hindered due to the presence of teeth and bone in the affected region. Oral malignant melanoma is aggressive, and the abundant blood supply of the oral cavity may permit blood vessel invasion and hematogenous dissemination early in the disease course. Compared with cutaneous and ocular melanoma, oral malignant melanoma has a poor prognosis with a reported 5-year survival rate of 10–25%.^1,4,5^ In the present case, the patient had no symptoms and the disease was not detected until pulmonary metastasis had been found. As a result, survival period was short, and the patient died after only one year.

For diagnosis of malignant melanoma, especially amelanotic melanoma, immunohistochemical examination is useful. Positive immunostaining for S100 and HMB45 is reported to have high sensitivity and specificity for malignant melanoma.^1,3,4^ In this case, the lung tumor was amelanotic, but was diagnosed as malignant melanoma on the basis of positive immunostaining for S100 and HMB45.

Surgery is the mainstay of treatment for oral melanoma.^1^ In the present case, we performed surgery for the oral lesion despite the advanced stage of disease with pulmonary metastasis, because no other distant metastasis was detected, resection of a solitary lung metastasis has been reported to improve the prognosis,^6–8^ and surgical resection of oral tumor may prevent any decrease in the quality of life due to pain or eating disorder resulting from tumor progression. This patient suddenly died due to bleeding from a pleural metastasis one year after surgery, but no recurrent tumor was found in the oral cavity and normal oral food intake was possible until the time of death.

The optimal postoperative treatment for malignant melanoma has not been determined.^5^ The present patient was 84 years old and he declined chemotherapy, being followed up without further treatment in spite of his advanced disease. Several reports have indicated that immunotherapy and chemotherapy are effective for malignant melanoma.^1,4^ It is expected that further improvements in the treatment of malignant melanoma will be made, such as targeted drug delivery directed against the cancer specific antigen(s).

## 4 Conclusion

We have presented a case of amelanotic melanoma of the lung that was proved as metastatic tumor from an oral malignant melanoma by detailed physical examination including the oral cavity. Oral malignant melanoma was very rare, but oral cavity should be examined when the pulmonary nodule was diagnosed as malignant melanoma.

## Conflict of interest

None declared.

## Funding

None.

## Ethical approval

Written informed consent was obtained from the patient for publication of this case series and accompanying images. A copy of the written consent is available for review by the Editor-in-Chief of this journal on request.

## Author contributions

Katsunari Matsuoka: data collection and writing the paper.
